# Rating of Perceived Exertion: A Large Cross-Sectional Study Defining Intensity Levels for Individual Physical Activity Recommendations

**DOI:** 10.1186/s40798-024-00729-1

**Published:** 2024-06-10

**Authors:** Maximilian Grummt, Lorena Hafermann, Lars Claussen, Carolin Herrmann, Bernd Wolfarth

**Affiliations:** 1 Department of Sports Medicine, Charité – Universitätsmedizin Berlin, Philippstr. 13 Haus 11, 10115 Berlin, Germany; 2 Institute of Biometry and Clinical Epidemiology, Charité – Universitätsmedizin Berlin, Corporate Member of Freie Universität and Humboldt-Universität zu Berlin, Berlin, Germany; 3 Institute of Sports Science, Humboldt-Universität zu Berlin, Berlin, Germany

**Keywords:** Rating of perceived exertion, RPE, Individualized intensity recommendation, Age, Sex, Type of ergometry, Cardiopulmonary fitness, Duration of exercise

## Abstract

**Background:**

Physical inactivity is a growing risk factor worldwide, therefore getting people into sports is necessary. When prescribing physical activity, it is essential to recommend the correct training intensities. Cardiopulmonary exercise testing (CPX) enables precise determination of individuals’ training intensities but is unavailable for a broad population. Therefore, the Borg scale allows individuals to assess perceived exertion and set their intensity easily and cost-efficiently. In order to transfer CPX to rating of perceived exertion (RPE), previous studies investigated RPE on specific physiological anchors, e.g. blood lactate (bLa) concentrations, but representativeness for a broad population is questionable. Some contradictory findings regarding individual factors influencing RPE occur, whereas univariable analysis has been performed so far. Moreover, a multivariable understanding of individual factors influencing RPE is missing. This study aims to determine RPE values at the individual anaerobic threshold (LT2) and defined bLa concentrations in a large cohort and to evaluate individual factors influencing RPE with multivariable analysis.

**Methods:**

CPX with bicycle or treadmill ergometer of 6311 participants were analyzed in this cross-sectional study. RPE values at bLa concentrations 2 mmol/l, 3 mmol/l, 4 mmol/l, and LT2 (first rise in bLa over baseline + 1.5 mmol/l) were estimated by spline interpolation. Multivariable cumulative ordinal regression models were performed to assess the influence of sex, age, type of ergometry, VO2max, and duration of exercise testing on RPE.

**Results:**

Median values [interquartile range (IQR)] of the total population were RPE 13 [11; 14] at 2 mmol/l, RPE 15 [13; 16] at 3 mmol/l, RPE 16 [15; 17] at 4 mmol/l, and RPE 15 [14; 16] at LT2. Main influence of individual factors on RPE were seen especially at 2 mmol/l: male sex (odds ratio (OR) [95%-CI]: 0.65 [0.587; 0.719]), treadmill ergometry (OR 0.754 [0.641; 0.886]), number of stages (OR 1.345 [1.300; 1.394]), age (OR 1.015 [1.012; 1.018]), and VO2max (OR 1.023 [1.015; 1.030]). Number of stages was the only identified influencing factor on RPE at all lactate concentrations/LT2 (3 mmol/l: OR 1.290 [1.244; 1.336]; 4 mmol/l: OR 1.229 [1.187; 1.274]; LT2: OR 1.155 [1.115; 1.197]).

**Conclusion:**

Our results suggest RPE ≤ 11 for light intensity, RPE 12–14 for moderate intensity, and RPE 15–17 for vigorous intensity, which slightly differs from the current American College of Sports Medicine (ACSM) recommendations. Additionally, we propose an RPE of 15 delineating heavy and severe intensity domain. Age, sex, type of ergometry, duration of exercise, and cardiopulmonary fitness should be considered when recommending individualized intensities with RPE, primarily at lower intensities. Therefore, this study can be used as a new guideline for prescribing individual RPE values in the clinical practice, predominantly for endurance type exercise.

**Supplementary Information:**

The online version contains supplementary material available at 10.1186/s40798-024-00729-1.

## Key Points

- To promote physical activity, the Borg Scale (6–20) is an easy and accessible tool for the recommendation of training intensities.
- Our results suggest RPE ≤ 11 for light intensity, RPE 12–14 for moderate intensity, and RPE 15–17 for vigorous intensity, and RPE 15 delineating heavy and severe intensity domain.
- In clinical routine, physicians and therapists should consider age, sex, type of ergometry, duration of exercise, and cardiopulmonary fitness as factors influencing RPE, especially with lower intensities.

**Supplementary Information:**

The online version contains supplementary material available at 10.1186/s40798-024-00729-1.

## Background

Promoting physical activity is more important than ever, as physical activity has decreased over the last decades - although it has an undeniable impact on human health [1–7]. International societies such as the World Health Organization (WHO) and the American College of Sports Medicine (ACSM) recommend at least 150 min moderate-intensity, 75 min vigorous-intensity, or 500–1000 metabolic equivalent (MET)∙min combined moderate-vigorous physical activity per week [8]. Resistance, flexibility, and neuromotor exercise training should be added with increasing age [9]. For physicians and therapists, getting people to exercise is a challenge, especially sedentary individuals. Therefore, it is essential to recommend the right intensity individually when they are willing to start physical activity as this contributes to motivation and persistence [10].

An effective way to classify exercise intensities and intensity prescription is by using exercise intensity domains, which divide exercise intensities into the moderate, heavy, and severe domain [11, 12]. Each domain is characterized by a specific physiological response, e.g. blood lactate (bLa): In the moderate domain, bLa remains at baseline level, whereas in the heavy domain, bLa rises above the baseline and reaches a steady state, and in the severe domain, bLa accumulates [11, 13–20]. A recent review by Jamnick et al. [11] examined anchors to produce consistent homogenous homeostatic perturbation and delineate these intensity domains. The authors concluded that submaximal anchors, i.e. lactate threshold 1 (LT1), gas exchange threshold (GET) and ventilatory threshold (VT) delineate the moderate and heavy domain, and critical power (CP) and critical speed (CS) delineates heavy and severe domain. It is advocated, that LT2 delineate the heavy and severe domain [11]. Whereas the method LT1 + 1.5 mmol/l was reported to estimate maximal lactate steady state (MLSS) validly, more than 30 methods exist to calculate LT2, which makes the evaluation of this anchor challenging [21]. Beyond that fixed bLa concentrations, such as 2.0 and 4.0 mmol/l, are proposed as demarcating the moderate/heavy and heavy/severe exercise domain, respectively [22–29]. In order to divide the training intensity into the three zones, cardiopulmonary exercise testing (CPX) with measurement, e.g. of bLa concentration, is the gold standard. CPX and bLa can also explain the physiological processes and shifts that occur during exercise at a given intensity. Despite CPX yielding much information, those diagnostics require personnel, technical, and financial resources. For some participants, collecting bLa can be uncomfortable due to its invasive nature. Thus, CPX is not available to every individual from a public health perspective.

The easiest way to assess and recommend exercise intensity is the individual’s subjective perception. Common used methods are Rating of Perceived Exertion (RPE) [30], Talk Test (TT) [31], and Feeling Scale (FS) [32]. All these scales offer the significant advantage of being easily explained, practicable in everyday life, and not requiring financial or human resources. Whereas there is limited research on intensity prescription with FS, a recent review demonstrated the validity of RPE and TT demarcating LT1/VT1 and LT2/VT2 [33]. Existing scales for RPE include the Borg RPE scale [34], Category Ratio-10 (CR-10) and Category Ratio-100 (CR-100) scale [35], and OMNI Perceived Exertion scale [36]. Unlike the TT with its variables “positive”, “equivocal”, and “negative”, RPE allows for quantifiable adjustments in intensity, facilitating more precise monitoring and progression of exercise regimens [37–43]. Probably the most common RPE scale is the Borg scale, with its counts between 6 and 20, which is a widely used tool in sports medicine/sports science [34, 44]. The ACSM [8] recommends an RPE between 9 and 11 for light intensity, RPE 12–13 for moderate intensity, and RPE 14–17 for vigorous intensity training, whereas Bok et al. [33] concluded RPE of 10–11 demarcating LT1 (moderate/heavy intensity domain) and RPE of 13–15 demarcating LT2 (heavy/severe intensity domain). The conclusion by Bok et al. may be reasonable based on the existing literature. However, the varied study populations and methodological approaches across the included studies must be noted (Table 1). There is still no consensus on the method to determine RPE values for bLa. Linear [40], quadratic [35, 45, 46], exponential regression [47], linear interpolation [26], and graphical analysis [48] were used. Furthermore, while Scherr et al. [45] is the only study examining a larger sample of 2560 participants, its representativeness for a broad population remains debatable (median age 28 years, > 70% male participants). Additionally, RPE values at bLa concentrations were calculated across the entire sample rather than individually. Also, RPE values were analyzed as a continuous variable. Even if the Borg scale was constructed to increase linearly with exercise intensity, Borg et al. stated that it does not create an interval scale [34]. Moreover, it is questionable if individuals can differentiate RPE values, e.g., between 15.3 and 15.8. Therefore, it is necessary to analyze RPE values as ordinal scaled.

**Table 1:** RPE (Borg Scale) values at defined lactate concentrations and individual lactate thresholds of previous studies

		2 mmol/l	LT1	3 mmol/l	LT2	4 mmol/l	Methods, population characteristics
Scherr et al. [45]	Total (n = 2560)	–	10.8 (± 1.8)	12.8 (± 2.1)*	13.6 (± 1.8)	14.1 (± 2.0)	Lactate thresholds calculation: LT1 first rise in bLa, LT2 = LT1 + 1.5 mmol/l; RPE bLa regression: quadratic; age (years): median 28 (IQR17-44); sex m/w: 70/30%; activity level: self-reported average PA min/week, sedentary when ACSM recommendations not met; type of ergometry: bicycle and treadmill
	Athletes (n = 1187)	–	10.4 (± 1.7)^1^	12.8 (± 1.9)*	13.5 (± 1.7)	14.1 (± 1.8)	
	Sedentary (n = 1195)	–	11.2 (± 1.7)^1^	12.7 (± 2.1)*	13.6 (± 1.8)	14.0 (± 2.1)	
	Bicycle (n = 1521)	–	11.2 (± 1.7)^2^	12.8 (± 2.1)*	13.6 (± 1.8)	14.1 (± 2.1)	
	Treadmill (n = 1039)	–	10.2 (± 1.7)^2^	–	13.6 (± 1.8)	14.1 (± 1.9)	
	Men (n = 1798)	–	10.9 (± 1.7)	12.8 (± 2.2)*	13.7 (± 1.7)	14.1 (± 1.9)	
	Women (n = 764)	–	10.7 (± 1.8)	12.9 (± 1.9)*	13.5 (± 1.8)	14.0 (± 2.0)	
Irving et al. [49]	Total (n = 36)	–	10.1 (± 0.4)	–	–	15.6 (± 0.4)	Lactate threshold calculation: LT1 first rise in bLa over baseline (min. 0.2 mM); RPE bLa regression: not specified; age (years): men 45.3 (± 3.8), women 46.0 (± 2.4); sex m/w: 28/72%; activity level: sedentary to light active (< 2times/week, self-reported); type of ergometry: treadmill
Abe et al. [47]	Untrained (n = 11)	–	11.2 (± 1.5)^3^	–	–	15.6 (± 2.1)	Lactate threshold calculation: LT1 log–log transformation; regression RPE/bLa: exponential; age (years): untrained 23.3 (± 2.9), distance runner 19.1 (± 1.0), race walker 19.2 (± 1.1); sex m/w: 0/100%; activity level: self-reported; type of ergometry: treadmill
	Distance runner (n = 15)	–	12.3 (± 1.6)^3^	–	–	16.7 (± 1.8)	
	Race walker (n = 6)	–	13.0 (± 1.6)^3^	–	–	16.9 (± 1.8)	
Hetzler et al. [50]	Bicycle (n = 29)	13.1 (± 2.1)	10.2 (± 2.2)	–	–	16.0 (± 2.3)	Lactate threshold calculation: LT1 first rise in bLa over baseline (min. 0.2 mM); RPE bLa regression: not specified; age (years): 31.5 (± 4.8); sex m/w: 100/0%; activity level: untrained (self-reported); type of ergometry: treadmill and bicycle (test/re-retest)
	Treadmill (n = 29)	13.8 (± 1.8)	10.8 (± 1.9)	–	–	16.2 (± 2.6)	
Demello et al. [51]	Sedentary men (n = 10)	–	13.5 (± 1.5)	–	–	–	Lactate threshold calculation: LT1 first rise in bLa over baseline; RPE bLa regression: not specified; age (years): 31.5 (± 4.8); sex m/w: 50/50%; activity level: self-reported (trained: 50 km/week running); type of ergometry: treadmill
	Sedentary women (n = 10)	–	12.9 (± 1.3)	–	–	–	
	Trained men (n = 10)	–	13.6 (± 2.1)	–	–	–	
	Trained women (n = 10)	–	13.5 (± 1.6)	–	–	–	
Hutchinson et al. [46]	Bicycle (n = 8)	–	est. 10	–	–	–	Lactate threshold calculation: LT1 log–log transformation, LT2 = LT1 + 1.5 mmol/l; RPE bLa regression: quadratic; age (years): 21 (± 3); sex m/w: 100/0%; activity level: recreationally active (self-reported); type of ergometry: bicycle and handcylce
	Handcycle (n = 8)	–	est. 10	–	–	–	
Rynders et al. [52]	Total (n = 148)	–	10.4 (± 2.0)	–	–	–	Lactate threshold calculation: LT1 first rise in bLa over baseline; RPE bLa regression: not specified; age (years): total 20.5 (± 13.9), old (< 50 years) 57.7 (± 6.7), young (18–35 years) 24.1 (± 3.8); sex m/w: 50/50%; activity level: untrained (self-reported, fitness level highest and lowest tertial according to VO2max); type of ergometry: bicycle
	Men (n = 74)	–	10.7 (± 1.9)	–	–	–	
	Women (n = 74)	–	10.1 (± 2.1)	–	–	–	
	Young (n = 120)	–	10.5 (± 2.0)	–	–	–	
	Old (n = 28)	–	9.8 (± 1.8)	–	–	–	
	Least fit (n = 50)	–	10.2 (± 2.0)	–	–	–	
	Most fit (n = 49)	–	10.8 (± 2.2)	–	–	–	
Held et al. [26]	Men (n = 319)	–	–	–	–	15.1 (± 1.9)	Lactate threshold calculation: not applicable; RPE/bLa regression: linear interpolation; age (years): women 22.7 (± 4.5), men 22.9 (± 5.5); sex m/w: 69/31%; activity level: squad athletes; type of ergometry: treadmill
	Women (n = 145)	–	–	–	–	14.9 (± 1.7)	
	10% worst women (n = n.s.)	–	–	–	–	13.2 (± 1.1)^4^	
	10% best women (n = n.s.)	–	–	–	–	16.1 (± 1.3)^4^	
	10% worst men (n = n.s.)	–	–	–	–	12.3 (± 1.8)^4^	
	10% best men (n = n.s.)	–	–	–	–	16.6 (± 1.1)^4^	

Values as mean (± standard deviation) unless otherwise stated. bLa blood lactate concentration, f female, LT1 lactate threshold 1, LT2 lactate threshold 2, m male, n.s. not specified, RPE rating of perceived exertion, *RPE values at lactate concentration 3 mmol/l for bicycle ergometry only. ^1^p < 0.05 athletes versus sedentary; ^2^p < 0.05 treadmill versus bicycle ergometry; ^3^p < 0.05 distance runner and race walker vs untrained; ^4^p < 0.001 10% worst and 10% best in women and men

Besides the general availability of intensity recommendations based on RPE, individual recommendations are still missing. Previous studies addressed this research question, but inconsistencies remain regarding the influence of various individual factors on RPE. Five studies focusing on fitness status revealed contradictory findings. Abe et al. [47] and Held et al. [26] reported higher RPE values among trained individuals. In contrast, Demello et al. [51] and Rynders et al. [52] found no significant difference in RPE between trained and untrained individuals, whereas Scherr et al. [45] even observed lower RPE values among trained subjects. The impact of ergometry type on RPE revealed mixed results, too. While Scherr et al. [45] reported higher RPE values during bicycle ergometry, Hetzler et al. [50] and Hutchinson et al. [46] reported no significant differences across different ergometry types. Regarding age, Scherr et al. [45] and Rynders et al. [52] observed no significant differences. It has to be noted that the influence of age has mainly been investigated through categorical grouping rather than with continuous variable analysis. Sex did not significantly influence RPE in previous research [26, 45, 51–54]. It is important to highlight that previous studies predominantly used univariable analyses to assess subgroup differences [26, 45–47, 50–54]. A comprehensive multivariable understanding of their individual effects on RPE is still lacking. Notably, the impact of exercise duration, in relation to the previously mentioned influencing factors, could confound previous findings and should, therefore, be adjusted for.

In conclusion, it is necessary to identify RPE values and individual influence factors within a broad study population. Moreover, there is the need to evaluate RPE values at physiological anchors on an ordinal scale and identify individual influencing factors using multivariable analysis, as we hypothesize that sex, age, type of ergometry, VO2max, and duration of exercise testing influence RPE. The selection of bLa as the physiological anchors for determination of RPE is based on their aforementioned wide use in exercise physiology and intensity recommendation, as well as their availability in a large data set of CPX, and their use in previous studies. Specifically, 2 mmol/l and 4 mmol/l are proposed to delineate the moderate/heavy and heavy/severe intensity domain, respectively, while the 3 mmol/l threshold reflects an intensity within the heavy intensity domain. Additionally, LT2 delineates the heavy and severe domain, whereas it is important for exercise control when prescribing intensities with RPE for a broad population, especially avoiding intensities too exhaustive.

Taking all of this into account, the aims of this study are

1. The determination of RPE values at individual anaerobic threshold (LT2) and defined bLa concentrations 2 mmol/l, 3 mmol/l, and 4 mmol/l with RPE as ordinal scaled and
2. The evaluation of the influence of sex, age, type of ergometry, VO2max, and duration of exercise testing on RPE with multivariable cumulative ordinal regression models.

## Material and Methods

### Study Design, Setting, Participants, Variables

In this cross-sectional study, we analyzed data of CPXs collected at the Department of Sports Medicine, Charité - Universitätsmedizin Berlin and Humboldt-Universität zu Berlin, Germany, from 2015 to 2022. The Department of Sports Medicine operates as an outpatient clinic in the regular German care system, accessible to all German (and even international) citizens seeking sports medical examination and CPX. The data set contained information about individual RPE and bLa values for each stage, bLa at the individual lactate thresholds LT1 and LT2, age, sex, BMI, weight, height, and type of ergometry (treadmill or bicycle). CPXs were exported pseudonymized from the department’s electronic system. Data sets were excluded if the following applied: (A) re-tests (only the first CPX for each participant was analyzed, when multiple CPXs of single participants were performed), (B) missing bLa, (C) missing RPE, (D) less than three stages in CPX, (E) other types of ergometry than bicycle and treadmill, and (F) implausible data. A flow chart showing the exclusion process is given in the online supplementary material (OSM) 1. This study was performed in line with the principles of the Declaration of Helsinki. Approval was granted by the Ethics Committee of Charité - Universitätsmedizin Berlin (EA2/121/23).

### Measures, Cardiopulmonary Exercise Testing

Weight and height were measured undressed (seca GmbH & Co. KG, Hamburg, Deutschland), and BMI was calculated as kg/m^2^. Participants performed graded CPX on bicycle (LC6 Monark, Vansbro, Sweden; ergoselect 100 K ergoline GmbH, Bitz, Germany) or treadmill (T170 h/p/cosmos, Traunstein, Germany; mercury h/p/cosmos, Traunstein, Germany). After initial clinical assessment, each exercise protocol was defined: bicycle ergometry starting with 10–100 W increasing 10–50 W every 3 min and treadmill ergometry beginning with 3–10 km/h increasing 1–2 km/h every 3 min [55]. Individualized protocols were set to avoid intensities too low/high initially. The exercise protocol and the 15 counts Borg scale were explained to each participant carefully. Participants were asked to rate their overall RPE according to Macora et al. [56] as “the conscious sensation of how hard, heavy, and strenuous the physical task is”. Before the CPX resting heart rate (HR), baseline bLa and blood pressure were measured. 12-lead ECG (cardio 110BT & cardio 300BT customed, Ottobrunn, Germany) and HR were recorded continuously during CPX. During the last 30 s of each stage, RPE and blood pressure (only with bicycle ergometry during exercise) were measured, and 20 μl of blood from the earlobe was taken. BLa was analyzed fully automatically (Biosen S-line EKFDiagnostics, Barleben, Germany). The participants were asked to reach maximal volitional exhaustion at the end of CPX. Termination criteria for CPX were cadence < 70/min in bicycle ergometry, leaving a predefined running zone on the treadmill, occurring symptoms such as angina pectoris, ECG changes (e.g., ST segment depression > 2 mV in chest leads and > 1 mV in extremity leads) and exceeding the individual blood pressure limit. The standardized room temperature was 19–20.5 °C. VO2max was calculated following ACSM recommendation [8, 57, 58]. LT1 was analyzed automatically as the first rise in bLa over baseline level and LT2 as bLa at LT1 + 1.5 mmol/l (as defined by Dickhuth et al.) with the software Ergonizer (5.0.1, Freiburg i. Brsg., Germany) [18, 55, 59–61]. Subjects with pre-existing conditions that did not allow maximal exercise load were excluded.

### Statistical Methods

All variables are described descriptively by providing mean and standard deviation (SD) for continuous variables and absolute and relative frequencies for categorical variables. Since a very high sample size is analyzed, we consider the normal distribution assumption to be valid. Furthermore, those descriptive characteristics are given stratified by sex, type of ergometry, and age groups.

**Fig. 1:**
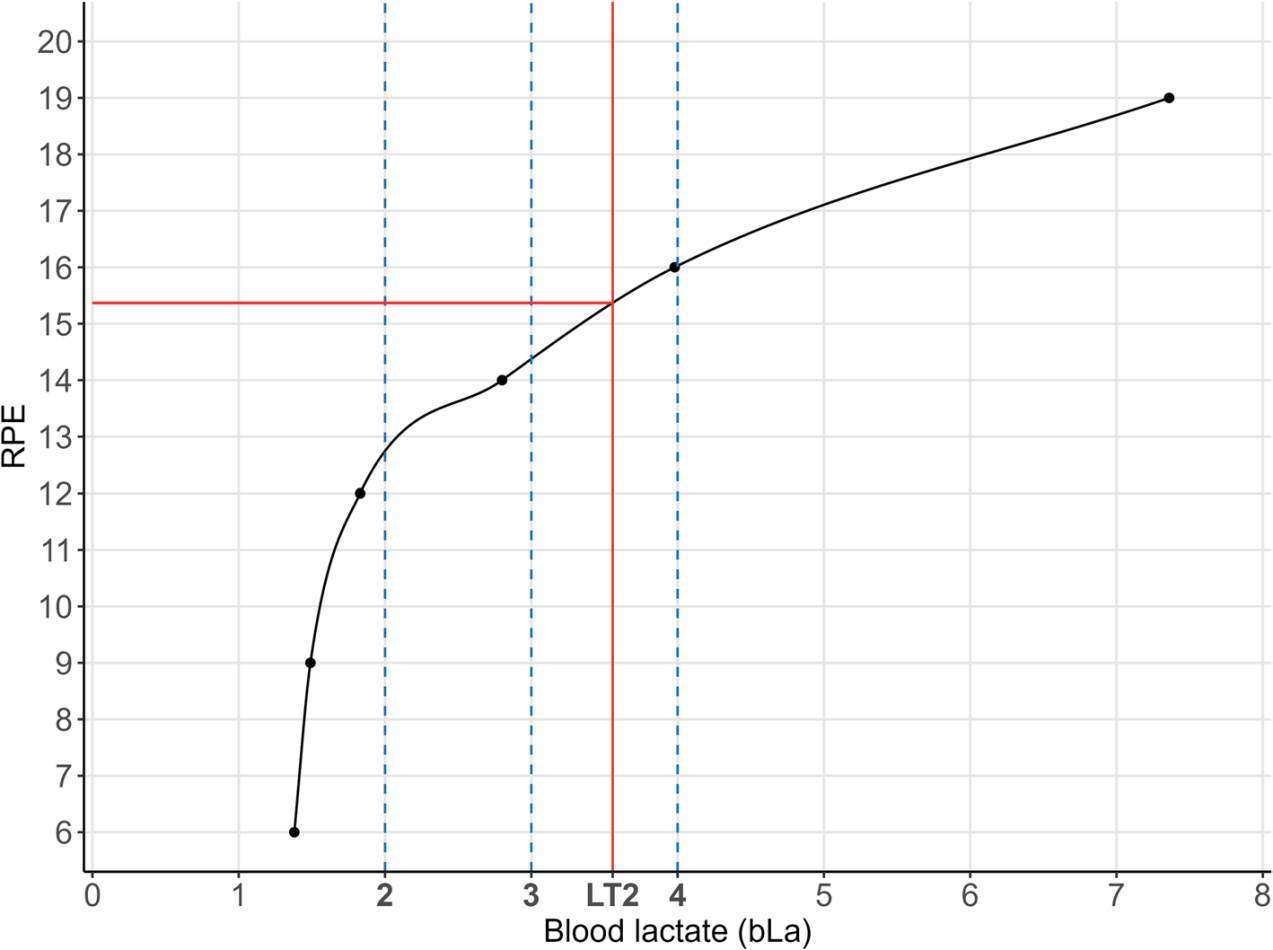
Illustration of the spline interpolation for RPE values at the specific bLa concentrations (mmol/l) and LT2. LT2 lactate threshold 2, RPE rating of perceived exertion

The first aim of this study was the determination of the individual RPE values at the bLa threshold LT2, 2 mmol/l, 3 mmol/l, and 4 mmol/l. Since only bLa values and RPE values measured at the specific CPX stages were available, but no RPE values at the specific bLa concentrations/threshold, spline interpolation was used to link these points to create an individual function for each participant as shown in Fig. 1. For the spline interpolation, cubic monotone Hermite splines were used as described by Fritsch et al. [62]. We set a requirement of at least three pairs of bLa and RPE values for the interpolation. Only three persons did not meet these criteria and were excluded from the analysis. After interpolation, the corresponding RPE values were then retrieved by the rounded predicted RPE value at the specific bLa threshold/concentration (LT2, 2 mmol/l, 3 mmol/l, and 4 mmol/l). The distribution of the resulting RPE values at the different threshold/concentrations is illustrated in barplots.

The second aim was to analyze the influence of sex, age, type of ergometry, VO2max, and number of stages during the CPX on the RPE values at the different bLa threshold/concentrations (LT2, 2 mmol/l, 3 mmol/l, and 4 mmol/l). Therefore, four multivariable cumulative ordinal regression models were built with RPE at LT2, and RPE at bLa 2 mmol/l, 3 mmol/l, and 4 mmol/l as outcome. All variables from “Material and Methods” section are included in the full model estimation (without further variable selection) as independent variables. Since the data set included no missing values, the complete data set could be used. Furthermore, the predictions for differing sex, ergometry type, and VO2max or age are visualized in Figures. For this illustration, the number of stages and the age or VO2max are defined as the mean value in this population.

For the analysis and figures the software R (version: 4.2.1) was used. For the spline interpolation the function *splinefun()* (R-package stats version 4.2.1) and for the ordinal regression the function *polr()* (R-package MASS version: 7.3) was used. The complete R-code is available in OSM 6.

## Results

### Participants, Descriptive Data

A primary data set of 10934 CPXs was included in this cross-sectional study. Of these, 4623 CPXs were excluded based on the exclusion criteria. Afterwards, a sample population of n = 6311 was available for statistical analysis. Baseline characteristics are shown in Table 2; the exclusion process is visualized in OSM 1. According to WHO recommendation, 92 (1.7%) adult participants were underweight (BMI < 18.5 kg/m^2^), 3270 (61.0%) had a normal BMI (18.5–24.9 kg/m^2^), 1542 (28.8%) were pre-obese (25–29.9 kg/m^2^), 337 (6.3%) had obesity class I (30–34.9 kg/m^2^), 100 (1.9%) had obesity class II (35–39.9 kg/m^2^), and 18 (0.3%) had obesity class III (≥ 40 kg/m^2^). A total of 3673 (58.0%) bicycle ergometries and 2640 (41.7%) treadmill ergometries were performed. Baseline characteristics categorized by age groups are available in the supplementary material (OSM 2).

**Table 2:** Baseline characteristics of the study population

	Total	Sex		Type of ergometry	
	(n = 6311)	Men (n = 3949)	Women (n = 2362)	Bicycle (n = 3672)	Treadmill (n = 2639)
Age, years	36.65 (16.68)	36.10 (16.74)	37.57 (16.55)	41.47 (17.32)	29.95 (13.07)
Sex, n (%)					
Male	3949 (62.6)			2203 (60.0)	1746 (66.2)
Female	2362 (37.4)			1469 (40.0)	893 (33.8)
Type of ergometry, n (%)					
Bicycle	3672 (58.2)	2203 (55.8)	1469 (62.2)		
Treadmill	2639 (41.8)	1746 (44.2)	893 (37.8)		
BMI, kg/m^2^, n (%)					
< 18.5	389 (6.2)	212 (5.4)	177 (7.5)	150 (4.1)	239 (9.1)
18.5–24.9	3845 (60.9)	2187 (55.4)	1658 (70.2)	1986 (54.1)	1859 (70.4)
25–29.9	1597 (25.3)	1228 (31.1)	369 (15.6)	1096 (29.8)	501 (19.0)
30–34.9	349 (5.5)	239 (6.1)	110 (4.7)	313 (8.5)	36 (1.4)
35–39.9	106 (1.7)	70 (1.8)	36 (1.5)	103 (2.8)	3 (0.1)
> = 40	25 (0.4)	13 (0.3)	12 (0.5)	24 (0.7)	1 (< 0.1)
Weight, kg	73.92 (16.99)	79.52 (16.61)	64.54 (13.02)	76.61 (17.46)	70.16 (15.55)
Height, cm	174.87 (11.43)	179.42 (10.62)	167.26 (8.25)	174.81 (10.62)	174.95 (12.47)
VO2max, ml/kg/min	38.64 (12.68)	41.88 (12.14)	33.24 (11.68)	31.40 (10.76)	48.72 (6.99)

Values are expressed as mean ± standard deviation unless otherwise indicated. BMI body mass index, VO2max maximal oxygen uptake

### RPE at LT2 and Defined Lactate Concentrations

**Fig. 2:**
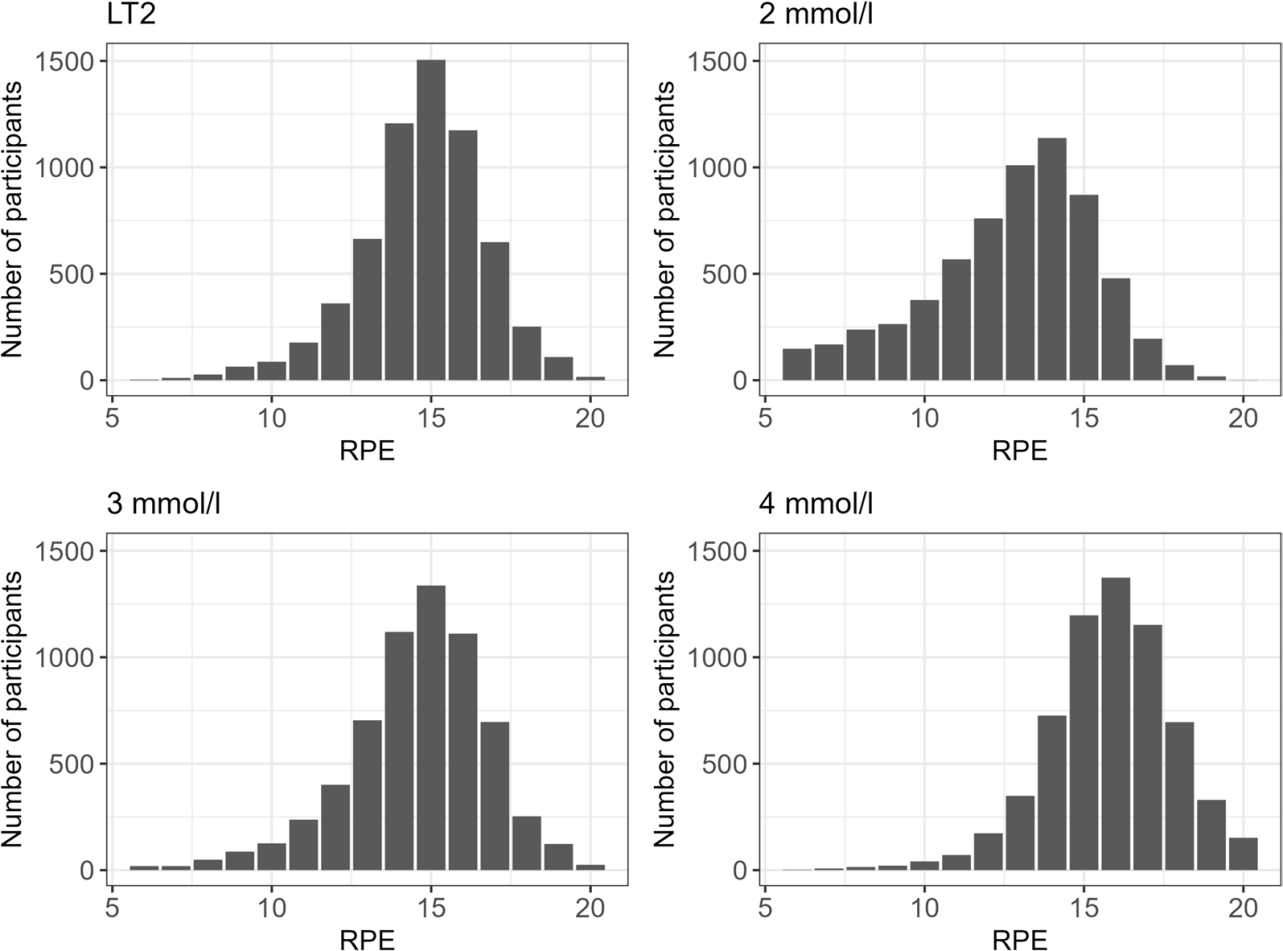
RPE distribution at defined bLa concentrations and LT2. LT2 lactate threshold 2, RPE rating of perceives exertion

Median and Interquartile range (IQR) of RPE values at LT2 and defined bLa concentrations in the total population were RPE 13 [11; 14] at 2 mmol/l, RPE 15 [13; 16] at 3 mmol/l, RPE 16 [15; 17] at 4 mmol/l, and RPE 15 [14; 16] at LT2. The distribution is visualized in Fig. 2. The mean value of bLa concentration for LT2 was 3.03 mmol/l (± 0.66).

### Multivariable Cumulative Ordinal Regression Models to Evaluate the Influence of Interindividual Differences on RPE

**Fig. 3:**
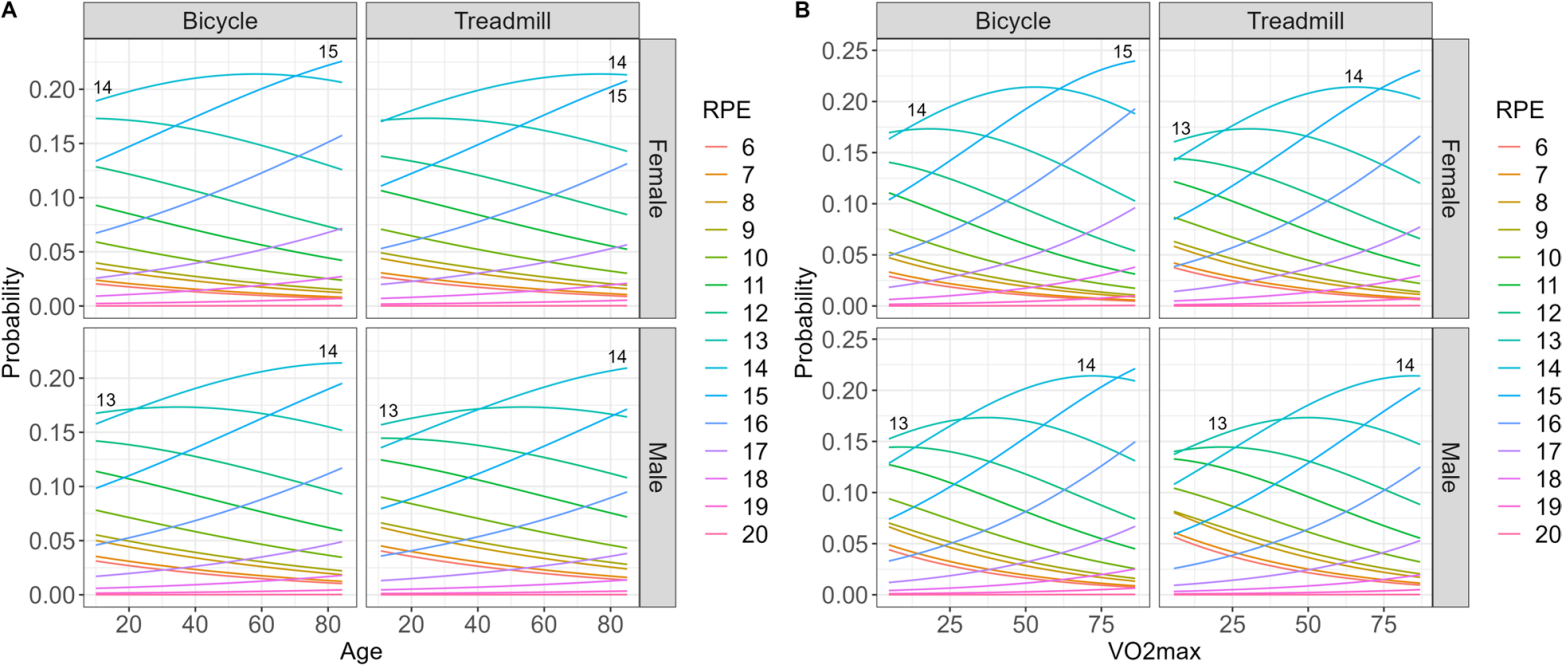
Predicted probabilities for RPE at 2 mmol/l in relation to the type of ergometry, sex and **A** age or **B** VO2max

Multivariable cumulative ordinal regression models show that individual factors influence RPE, especially at 2 mmol/l (Table 3). At this bLa concentration, male sex (OR 0.65 95%-CI [0.587; 0.719]), treadmill ergometry (OR 0.754 [0.641; 0.886]), and number of stages (OR 1.345 [1.300; 1.394]) show a greater effect than age (OR 1.015 [1.012; 1.018]) and VO2max (OR 1.023 [1.015; 1.030]). However, the OR regarding age and VO2max reflecting a difference of 1 year or 1 ml/kg/min, respectively. An increase of 1 year or 1 ml/kg/min is therefore rather negligible, but changes can be relevant with a greater age/fitness difference. Therefore, Fig. 3 visualizes the predicted probabilities for RPE at 2 mmol/l in relation to type of ergometry, sex, and VO2max or age, respectively.

**Table 3:**
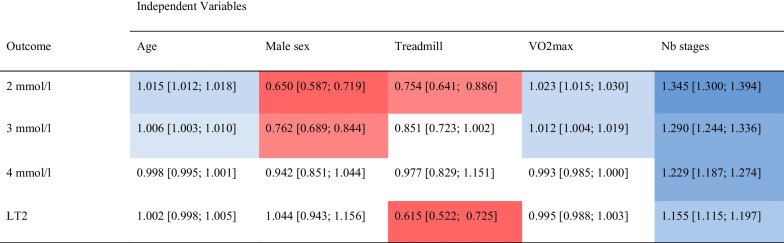
Multivariable cumulative ordinal regression models to predict the risk of individual factors to influence RPE at defined lactate concentrations and individual lactate threshold

Values are expressed as OR (95%-CI). LT1 lactate threshold 1, LT2 lactate threshold 2, Nb number, RPE rating of perceived exertion, VO2max maximal oxygen uptake, red indicate lower ORs and blue indicate higher ORs, the color intensity refers to the strength of the effect, no color indicate ORs near 1

From a practical perspective, RPE in relation to age, sex, and type of ergometry should be highlighted, as no additional measured variable is needed. For example, at 2 mmol/l, a 20-year-old man on the treadmill should be recommended an RPE of 13, while a 75-year-old woman on the bicycle should be recommended an RPE of 15. If focusing on age difference for the same type of ergometry and sex at 2 mmol/l: For men on the treadmill under 40 years of age, an RPE of 13, and over 40 years of age, an RPE of 14 should be recommended. This cross-over can be seen for women on the bicycle at the age of approx. 70 years (RPE 14 vs. 15) and for men on the bicycle at the age of approx. 20 years (RPE 13 vs. 14). Regarding VO2max, the following relevant cross-over at 2 mmol/l can be seen: for men on the treadmill with VO2max approx. 40 ml/min/kg (RPE 13 vs. 14), for men on the bicycle with VO2max approx. 30 ml/min/kg (RPE 13 vs. 14), for women on the treadmill with VO2max approx. 20 ml/min/kg (RPE 13 vs. 14), and for women on the bicycle with VO2max at approx. 62 ml/min/kg (RPE 14 vs. 15).

At 3 mmol/l the ORs for age (OR 1.006 [1.003; 1.010]), VO2max (OR 1.012 [1.004; 1.019]), male sex (OR 0.762 [0.689; 0.844]), and number of stages (OR 1.290 [1.244; 1.336]) decreases, whereas type of ergometry is not significant any more. At this lactate concentration the predictions for differing sex, ergometry type, and VO2max or age show no clinical relevant influence on RPE (RPE 15) (OSM 3.1 and 4.1). At 4 mmol/l, RPE differences were associated only with the number of stages (OR 1.229 [1.187; 1.274]). At LT2, RPE differences were associated with treadmill ergometry (OR 0.615 [0.522; 0.725]) and number of stages (OR 1.155 [1.115; 1.197]). Probability plots for differing sex, ergometry type, and VO2max or age at 4 mmol/l (RPE 16) and LT2 (RPE 15) are available in the supplementary material (OSM 3.2, 3.3, 4.2, 4.3).

As seen above, number of stages is the only influencing factor on RPE at all lactate concentrations/LT2. Using the prediction plots for sex, type of ergometry, and number of stages, the following cross over can be seen at 2 mmol/l (OSM 5.1): the first between approx. 5–7 stages (RPE 13 vs. 14) and the second approx. 8-est. 11 stages (RPE 14 vs. 15). As OR for sex and type of ergometry are decreasing with 3 and 4 mmol/l, the span of the number of stages at the crossing points decreases as well (OSM 5.2 and 5.3): from approx. 4–6 stages (RPE 14 vs. 15) and approx. 8–10 stages (RPE 15 vs. 16) at 3 mmol/l to approx. 5 stages (RPE 15 vs. 16) and approx. 9 stages (RPE 16 vs. 17) at 4 mmol/l. As the influence of treadmill ergometry on LT2 is significant we saw the following crossing points with no influence of sex: for treadmill at approx. 5 stages (RPE 14 vs. 15) and for bicycle at approx. 10 stages (RPE 15 vs. 16) (OSM 5.4). Each stage corresponds to a time of 3 min during the CPX. It is important to note that the intensity increases with each stage, so that an increased number of stages is also accompanied by an increase in intensity.

## Discussion

Our results present RPE values at different physiological anchors for a broad population resulting from the analysis of CPX of 6311 participants. Moreover, this study demonstrates that RPE is influenced by individual factors, especially at low intensities, which should be recognized when prescribing physical activity.

### RPE Recommendations for a Broad Population

Our results suggest RPE 13 [11; 14] at 2 mmol/l, RPE 15 [13; 16] at 3 mmol/l, RPE 16 [15; 17] at 4 mmol/l, and RPE 15 [14; 16] at LT2. This is consistent with the results for the total sample from Irving et al. [49] (4 mmol/l: 15.6 ± 0.4). Other previous studies reported RPE values only for subgroups, with confirming results from Abe et al. [47] (4 mmol/l: 15.6 (± 2.1) (untrained group)), Held et al. [26] (4 mmol/l: 10% best women 16.1 ± 1.3) and Hetzler et al. [50] (bicycle: 13.1 ± 2.1 at 2 mmol/l, 16.0 ± 2.3 at 4 mmol/l; treadmill 16.2 ± 2.6 at 4 mmol/l), whereas also lower and higher RPE values for subgroups were reported (see Table 1). More importantly and in contrast to the results from the largest study cohort so far by Scherr et al. [45], we consistently observed higher RPE values by 1–2 (rounded) at 3 mmol/l, 4 mmol/l, and LT2 (2 mmol/l was not reported). We think that variations in RPE derive primarily from differing methodological approaches and study populations. We calculated RPE at bLa anchors using spline interpolation on an individual basis and believe that this method is more accurate for the relationship between RPE and bLa rather than quadratic regression for the whole sample size, which was used by Scherr et al. [45]. Moreover, our study included more than twice the number of participants with a higher average participant age. To underline the validity of our results for a broad population, we compared our population with the European reference population. According to current data, the mean age in the EU is 44.4 years, in contrast to 36.7 years in our population [63]. Comparing the distribution of the BMI, we observed a higher proportion of participants in the “normal weight” range (BMI 18.5–24.9 kg/m^2^) (60.9% Charité Sports Medicine vs. 45.8% EU), as well as lower proportions in the pre adiposity range (BMI 25–29.9 kg/m^2^) (25.3% Charité Sports Medicine vs. 35.2% EU) [64]. The relative frequency of obesity grade 1 and higher (BMI over 30 kg/m^2^) was also lower in our study population (7.6%) than in the EU (16.0%) [64]. Although our population was younger and leaner, we see our results as transferable to a broad population. As mentioned before, we believe that our results more accurately represent a broader population compared to previous studies. Taking all of this into account and with substantial differences, especially between Scherr et al. [45] and our results, current intensity recommendations based on RPE should be redefined. We suggest RPE ≤ 11 for light intensity, RPE 12–14 for moderate intensity, and RPE 15–17 for vigorous intensity. While we do not propose changes for light intensity, as we did not calculate LT1, our findings support an RPE of 15 delineating heavy and severe intensity domain. Consequently, regarding the ACSM recommendations, we suggest increasing RPE values for moderate and vigorous intensity.

### Influencing Factors on RPE

It is important to understand the neuropsychological mechanisms involved in generating RPE to discuss individual influencing factors on RPE. While the afferent feedback theory is a common theory [34, 65], a recent meta-analysis by Bergevin et al. [66] concluded that afferent feedback from exercising muscle (transmitted through group III/IV muscle afferents) is not the neurophysiological signal generating perceived exertion. Moreover, it supports the model of corollary discharge [67]. This model proposes that efferent copies of the motor command from premotor and motor areas are processed in sensory areas of the cerebral cortex, resulting in the generation of perceived exertion [67, 68]. Although in this model, the afferent feedback does not generate perceived exertion, alterations in muscular force production capacity (e.g. affected by neuromuscular fatigue) and corticospinal excitability can indirectly influence perceived exertion via alterations of the central motor command to sustain performance output [68, 69].

Our results indicate higher RPE values with increasing VO2max and age at 2 mmol/l and 3 mmol/l. This suggests that participants with increasing cardiorespiratory fitness and/or age should consider higher RPE values at 2 mmol/l and 3 mmol/l, whereas from a practical perspective a relevant difference can be only seen at 2 mmol/l (see Fig. 3). Cardiopulmonary fitness deteriorates with increasing age [70] due to changes in cardiovascular [71–73], musculoskeletal [74, 75] and respiratory [76, 77] function. A reduction of maximal stroke volume, HR, and av-oxygen-difference result in deterioration of cardiovascular efficiency [71–73]. Reduced muscle volume, quality, function and total mitochondria mass change muscle contraction efficiency and energy use [74, 75]. Additionally, changes in pulmonary function in older adults result in expiratory airflow limitation and an increase in work of breathing [76]. Corollary discharges are not limited to the motor command of movement execution, e.g. the limbs, but also to the motor command of the respiratory muscles [67]. These factors might explain higher RPE with increasing age due to a higher magnitude of the motor command to sustain the same neuromuscular output. A recent meta-analysis by Shah et al. [78] evaluated the influence of age on corticospinal excitability. Whereas they stated that reduction in corticospinal excitability is possible, they see limitations in the conclusion based on heterogeneities within and between analyzed studies. Other factors, such as age-related impairment in thermoregulation, cognitive function, and psychological aspects should also be noted. Impaired heat loss rate through changes in skin vasodilation and decrease of evaporative rate leads to alterations in thermoregulation, which exposes elderly more susceptible to hyperthermia [79–82], which can impair neuromuscular function [83, 84]. Impaired cognitive function showed higher RPE in older age [85], whereas positive/negative expectations regarding aging and aging self-stereotypes could psychologically influence RPE [86–88], but studies directly evaluating this influence on RPE at bLa levels are lacking. Taking all of this into account, age-related influence on RPE could be generated by elevation of the motor command and/or reduction of corticospinal excitability multifactorial. However, these differences might be only present with lower intensities and resolve with higher intensities reflected by higher bLa concentrations. Further studies should investigate the aforementioned age-related changes and directly evaluate the underlying mechanisms why they are present at low intensities. Our results add to the current knowledge, as previous studies reported no influences of age on RPE when evaluating RPE at specific bLa concentrations [45, 52]. Both studies evaluated the influence of age on RPE within age grouping and univariable analysis, probably underestimating age-related influences.

VO2max changes are seen in the literature as partly genetic but mainly due to training history [89, 90]. Long-term adaptions to endurance training results especially in cardiovascular, respiratory, and metabolic adaptions, most important by elevating maximal cardiac output, perfusion capacity of the muscle, and size and number of mitochondria, the latter increasing activity of oxidative enzymes [91–93]. VO2max can be trained at any age [94, 95] and reflects individuals’ fitness independently, where people with greater cardiorespiratory fitness gain a right shift in the bLa curve. Consequently, people with a higher level of fitness can realize higher workloads at the same bLa concentrations/thresholds compared to less fit people. This might result in higher RPE values due to a higher magnitude of the motor command, again this might be only present at lower intensities. Confirming our results, the study by Abe et al. reported significantly lower RPE for untrained (11.2 ± 1.5) compared to distance runners (12.3 ± 1.6) or race walkers (13.0 ± 1.6) at lower intensities (LT1), but not at 4 mmol/l. Results by Held et al. [26] support our finding in principle that fitter people report higher RPE, but this is contrary to us at 4 mmol/l (not reported at other bLa anchors). Interestingly, the RPE difference between the 10% best and 10% worst in this study was 2.9 for women and even 4.3 for men. Considering more closely, this might be caused by univariable analysis and exercise protocol, starting treadmill ergometry with a minimum speed of 2.0 m/s (7.2 km/h). Accompanying with higher workloads at same bLa anchors with higher fitness, longer duration of exercise before reaching bLa anchors could especially overestimate their findings substantially. Therefore, we adjusted in our multivariable model for number of stages, excluding the duration of exercise as a confounder for our finding for VO2max. Scherr et al. [45] reported opposite results with lower RPE for athletes compared to sedentary at LT1, but with no differences at 3 mmol/l, 4 mmol/l, and LT2. Additionally, Demello et al. [51] and Rynders et al. [52] saw no differences in terms of fitness at all. It remains unclear why results by Scherr et al. [45] showed lower RPE with higher fitness at lower intensities, as it is opposite to our results, and an extensive discussion of their results is missing. It is important to note that the categorization of fitness levels was conducted by self-reporting in previous studies. Therefore, we evaluated fitness level with VO2max, analyzing this influence on RPE independently of grouping and believe this approach enables an objective view on this influencing factor.

Our results propose lower RPE values at 2 mmol/l and 3 mmol/l for men. This could be attributed to multifactorial sex differences. Regarding substrate metabolization, women have a higher availability of circulating and muscular plasma fatty acid and have higher muscle insulin sensitivity [96, 97]. In combination with the influence of 17-beta-estradiol, the oxidation of fatty acids is increased while glycogen stores are spared, especially at low to moderate exercise intensities [98, 99]. In addition, women have a greater proportion area of type I muscle fibers, greater capillarization, and lower glycolytic enzyme activity [97, 100]. Therefore, more lipids and fewer carbohydrates are utilized, leading to lower respiratory exchange ratios in women during the aforementioned intensities [98]. Transferring these physiological sex differences to our observations on RPE, the enhanced fat oxidation in women might result in lower lactate production at low to moderate intensities. Therefore, a higher magnitude of the motor command might be necessary for women to realize a higher relative workload to achieve the same bLa anchors at 2 and 3 mmol/l compared to men, resulting in higher RPE. A recent study by Delp et al. [101] showed that women reported higher RPE in early follicular phase compared to ovulation and mid-luteal phase during CPX, underlying a potential influence of the menstrual cycle and hormonal changes on RPE. However, an understanding of the underlying mechanism on the influence of RPE and especially corollary discharges is missing. As we do not have information about the menstrual cycle in our data, we cannot rule out a potential confounder of this, whereas we then would expect an influence at higher bLa concentrations as well. Our findings are in contrast to the current knowledge, while previous studies reported no sex differences at any bLa concentration/thresholds or other physiological variables [26, 45, 51–54]. Our multivariable approach might reveal the influence of sex on RPE compared to previous studies, while physiological sex differences give a reasonable explanation for our results. Further research should evaluate the influence of sex differences with other physiological anchors than bLa and underlying mechanism of the hormonal/menstrual cycle influencing RPE.

We saw lower RPE values for treadmill than for bicycle ergometry at 2 mmol/l and LT2. The differences in physiological responses between cycling and running may derive from multifactorial mechanisms. Evidence shows that lower VO2max in cycling is associated with less muscle mass engaged and lower cardiac output, influenced by lower stroke volume and alterations in peripheral blood flow [102–104]. It is reported that delta efficiency is higher in running than in cycling, suspected by alterations in movement patterns/biomechanics [105], and ventilation is more impaired in cycling than running [104, 105]. Compared to our results, higher RPE for bicycle ergometry could be explained by alterations of the aforementioned differences and more localized muscle use in cycling compared to running. This might lead to a higher magnitude of the motor command, but only present at 2 mmol/l and LT2. Regarding alterations in peripheral blood flow, a delayed onset of bLa in peripheral capillary collection point of the earlobe might be influential. This could be addressed in further studies as well as the underlying mechanism of type of ergometry influencing RPE only at 2 mmol/l and LT2. Additionally, bLa at LT2 was slightly lower for treadmill vs. bicycle (2.85 vs. 3.16 mmol/l), which can also be influential at LT2 and might overestimate the influence at LT2, whereas at 3 mmol/l we saw statistically no influence of type of ergometry on RPE (OR 0.851 [0.723; 1.002]). Our findings confirm with Scherr et al. [45], who reported higher RPE values for bicycle ergometry at lower intensity (LT1), but saw no differences at 4 mmol/l and LT2 (2 mmol/l and 3 mmol/l not reported). As bLa at LT2 shows differences in our study and Scherr et al. did not state their corresponding bLa at LT2, the comparison of results is challenging. Moreover, they did not discuss the influence of type of ergometry extensively in their study. In contrast, Hetzler et al. [50] and Hutchinson et al. [46] reported no differences in terms of type of ergometry at all. Whereas both studies accompanied a relatively small sample size, Hutchinson et al. compared bicycle and handcycle, which is out of the scope of our study.

Number of stages as an indicator of exercise duration was the only confounding variable, which in this study showed an influence on higher RPE with more stages over all bLa anchors. Therefore, it underlines the importance of a multivariable approach and duration of exercise as confounding variable for RPE, as the above discussed influencing factors can lead to alterations in exercise duration during CPX their self. Exercise duration can lead to alterations in neuromuscular fatigue, affecting muscle force production and corticospinal excitability, and therefore influencing corollary discharges, and explaining our findings [66, 68, 106, 107]. Consistent with the study by Jesus et al. [108], they showed increased session RPE (sRPE) after 30 compared to 15 min after moderate and strong intensity, correlated to RPE of 3 and 5 on the CR-10 scale. Contrary to our findings, they did not find differences with weak intensity (2 on CR-10 scale). Consequently, our study suggests that higher RPE values with longer duration, also but not limited to lower intensities, should be considered. In addition to Jesus et al. [108], our results suggest an immediate influence on RPE at any time during exercise compared to session RPE reporting retrospectively perceived exertion. As mentioned earlier, intensity is rising with each stage during CPX. It is therefore inherently difficult to multiply the number of stages by 3 min in order to define the total exercise duration, as the maximum load is reached at the last stage rather than a constant intensity.

### Limitations

The main limitation of this study is its retrospective cross-sectional design. As data were generated pseudonymized from the institute’s clinical information system, no information about participants’ possible conditions was available. It should be noted that the subjects, i.e., those in routine care at the university sports medicine outpatient clinic, tend to aspire to an active lifestyle and are more active in sports. Besides, high quality requirements for CPX analysing software and personnel, individual user, or technical errors cannot be ruled out with this high number of data sets and the large time period. As only one CPX per participant was included in the analysis to exclude multiple testing, reproductively of bLa responses with re-tests were not possible. The CPXs were performed on two bicycle and treadmill ergometry models. Although we see this as a minor data quality limitation, using one model each would be associated with even higher standardization. In addition, the CPX took place at different times due to consulting hours. As RPE was calculated at bLa anchors derived from treadmill/bicycle ergometry, our results cannot be transferred to resistance type exercise. For the calculation of LT2, we used the calculation according to Dickhuth et al. [18]. Other bLa threshold models were not considered in this study. Spline interpolation was chosen for the regression of RPE values and bLa concentrations. Whether this method of the true RPE-bLa curve is most accurate has yet to be conclusively determined. An inaccuracy cannot be ruled out. From a practical perspective, even one point difference on the Borg scale can be challenging to specify accurately. Therefore, our results can be used as a practical tool for a broad population, but in some cases, it might be necessary to receive individual guidance from physicians and therapists. This is underlined by the IQRs ranging from 2–3 RPE values. Finally, we have evaluated the influence of individual factors on RPE at bLa anchors, whereas bLa may only be one factor that alters RPE. Other information like HR, oxygen uptake, respiratory/ventilatory rate, blood/muscle pH, mechanical strain, muscle damage, core temperature, carbohydrate availability, anthropometric parameters, and skin temperature, as well as mental fatigue, could have an influence, too, and should be considered when recommending physical activity.

## Conclusion

Our study enables the recommendation of exercise intensities with RPE for a broad population. In contrast to the current knowledge, higher RPE for moderate/vigorous intensity and heavy/severe intensity domain should be considered, respectively. Our results suggest RPE ≤ 11 for light intensity, RPE 12–14 for moderate intensity, and RPE 15–17 for vigorous intensity, which slightly contrasts with the current ACSM recommendations. Additionally, we propose an RPE of 15 delineating heavy and severe intensity domain. Multivariable analysis reveals individual factors influencing RPE, especially at lower intensities. Increasing age, higher fitness, and female sex leads to higher RPE at 2 mmol/l and 3 mmol/l, with a practical relevance only at 2 mmol/l. Treadmill ergometry leads to lower RPE at 2 mmol/l and LT2, whereas differences in bLa concentration at LT2 might overestimate the influence of ergometry type at LT2. Number of stages was the only variable influencing RPE at all bLa anchors. This might be trivial as neuromuscular fatigue increases with longer exercise duration, the more it is important to be adjusted for in multivariable analysis to exclude exercise duration as a confounder on other influencing factors. The possibility of including individual factors influencing RPE investigated in this study represents a new aspect for clinical routine. The predicted probability plots as provided in this study can be used as a new and valid tool for prescribing individual RPE values in the clinical practice. It can help physicians and therapists developing individual training programs to effectively promote exercise and health. Moreover, it allows individuals to tailor their workouts to their specific goals, ensuring that they are working at an appropriate and safe intensity.

### Supplementary Information

Online Supplementary Material.

R-Code.

R-Session Information.

## Data Availability

The datasets generated during and/or analyzed during the current study are available from the corresponding author on reasonable request.

## Abbreviations

ACSM: American College of Sports Medicine
bLa: Blood lactate
BMI: Body mass index
CP: Critical power
CPX: Cardiopulmonary exercise testing
CR: Category ratio
CS: Critical speed
LT: Lactate threshold
MET: Metabolic equivalent
MLSS: Maximum lactate steady state
RPE: Rating of perceived exertion
VO2max: Maximum oxygen uptake
VT: Ventilatory threshold
WHO: World Health Organization

## Code Availability

The complete R-code is available in Online Supplementary Material 6 (file 2).
