# Supplementary material for: Rating of Perceived Exertion: A Large Cross-Sectional Study Defining Intensity Levels for Individual Physical Activity Recommendations
Source: Sports Med Open. 2024 Jun 10;10:71. doi: 10.1186/s40798-024-00729-1 (PMC11164849; doi:10.1186/s40798-024-00729-1)
Supplement: Supplementary file 1 — Online Supplementary Material. [file 40798_2024_729_MOESM1_ESM.pdf]

Rating of Perceived Exertion – A large cross-sectional study defining intensity levels for individual physical activity recommendations

Grummt M[1*], Hafermann L[2], Claussen L[3], Herrmann C[2], Wolfarth B[1,3]

[1] Department of Sports Medicine, Charité – Universitätsmedizin Berlin, Berlin, Germany

[2] Institute of Biometry and Clinical Epidemiology, Charité – Universitätsmedizin, corporate member of Freie Universität and Humboldt-Universität zu Berlin, Berlin, Germany

[3] Institute of Sports Science, Humboldt-Universität zu Berlin, Berlin, Germany

*** Corresponding author:**

Dr. Maximilian Grummt, MD

Department of Sports Medicine

Charité – Universitätsmedizin Berlin

Philippstr. 13 Haus 11, 10115 Berlin, Germany

maximilian.grummt@charite.de

+49 30 2093 46026

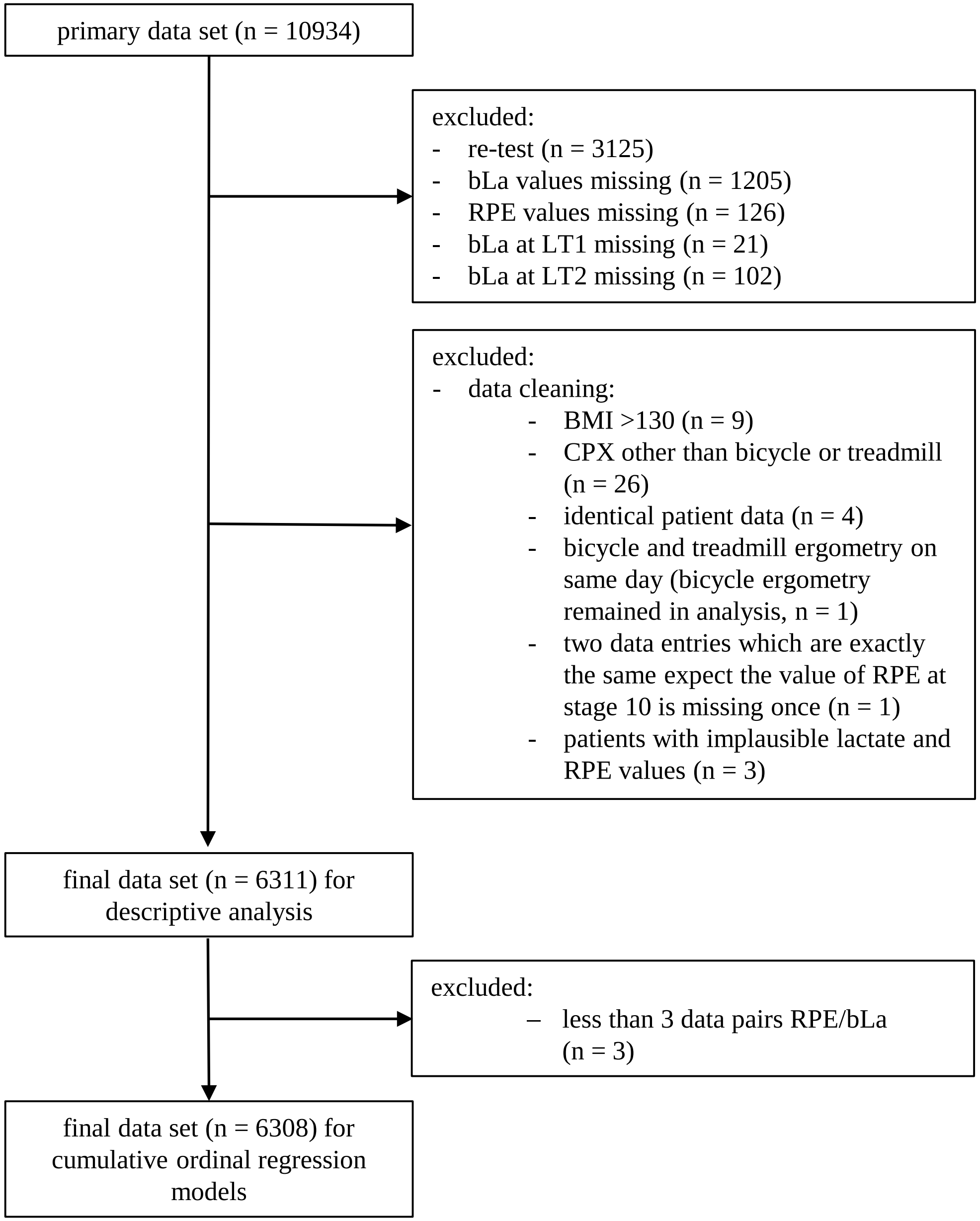

OSM 1 Flow chart of the exclusion process. bLa blood lactate, CPX cardiopulomary exercise testing, LT1 lactate threshold 1, LT2 lactate threshold 2, RPE rating of perceived exertion

OSM 2 Baseline characteristics of the study population categorized by age groups

		Age Group (years)			
		≤ 20 (n = 1228)	21 – 40 (n = 2620)	41—60 (n = 1807)	> 60 (n = 658)
Sex, n (%)	Male	819 (66.7)	1634 (62.4)	1096 (60.7)	402 (61.1)
	Female	409 (33.3)	986 (37.6)	711 (39.3)	256 (38.9
Type of ergometry, n (%)	Treadmill	753 (61.3)	1306 (49.8)	532 (29.4)	49 (7.4)
	Bicycle	475 (38.7)	1314 (50.2)	1275 (70.6)	609 (92.6)
BMI, kg/m^2, n (%)	<18.5	304 (24.8)	61 (2.3)	19 (1.1)	5 (0.8)
	18.5 – 24.9	784 (63.8)	1816 (69.3)	986 (54.6)	259 (39.4)
	25 – 29.9	113 (9.2)	634 (24.2)	576 (31.9)	276 (41.9)
	30 – 34.9	14 (1.1)	80 (3.1)	161 (8.9)	94 (14.3)
	35-39-9	6 (0.5)	21 (0.8)	58 (3.2)	21 (3.2)
	>=40	7 (0.6)	8 (0.3)	7 (0.4)	3 (0.5)
Weight, kg		63.05 (19.03)	75.06 (14.64)	78.34 (16.20)	77.48 (15.08)
Height, cm		170.99 (15.73)	177.32 (9.95)	175.51 (9.40)	170.55 (9.14)
VO2max, ml/kg/min		44.48 (9.36)	43.20 (11.07)	33.71 (11.46)	23.09 (8.75)

Values are expressed as mean ± standard deviation unless otherwise indicated. BMI body mass index, VO2max maximal oxygen uptake

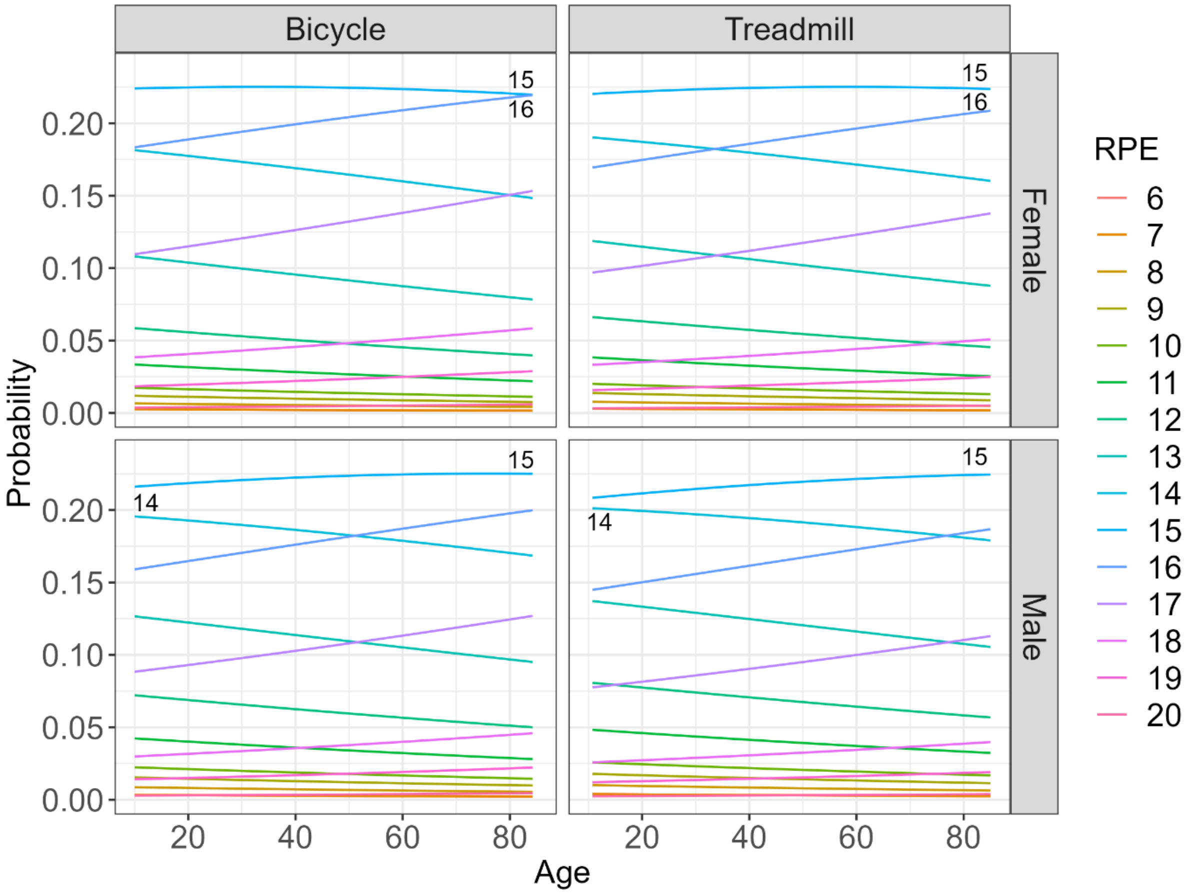

OSM 3.1 Predicted probabilities for RPE at 3 mmol/l in relation to the type of ergometry, gender and age

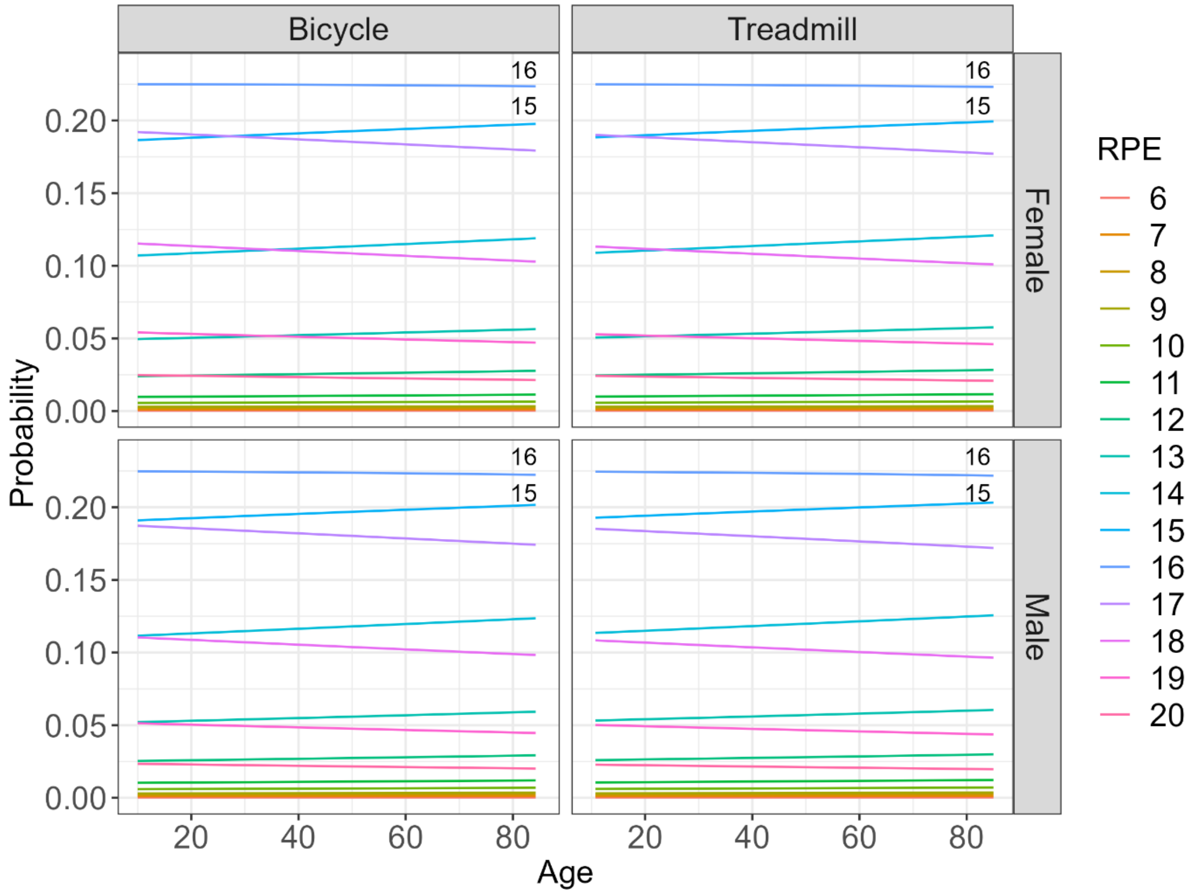

OSM 3.2 Predicted probabilities for RPE at 4 mmol/l in relation to the type of ergometry, gender and age

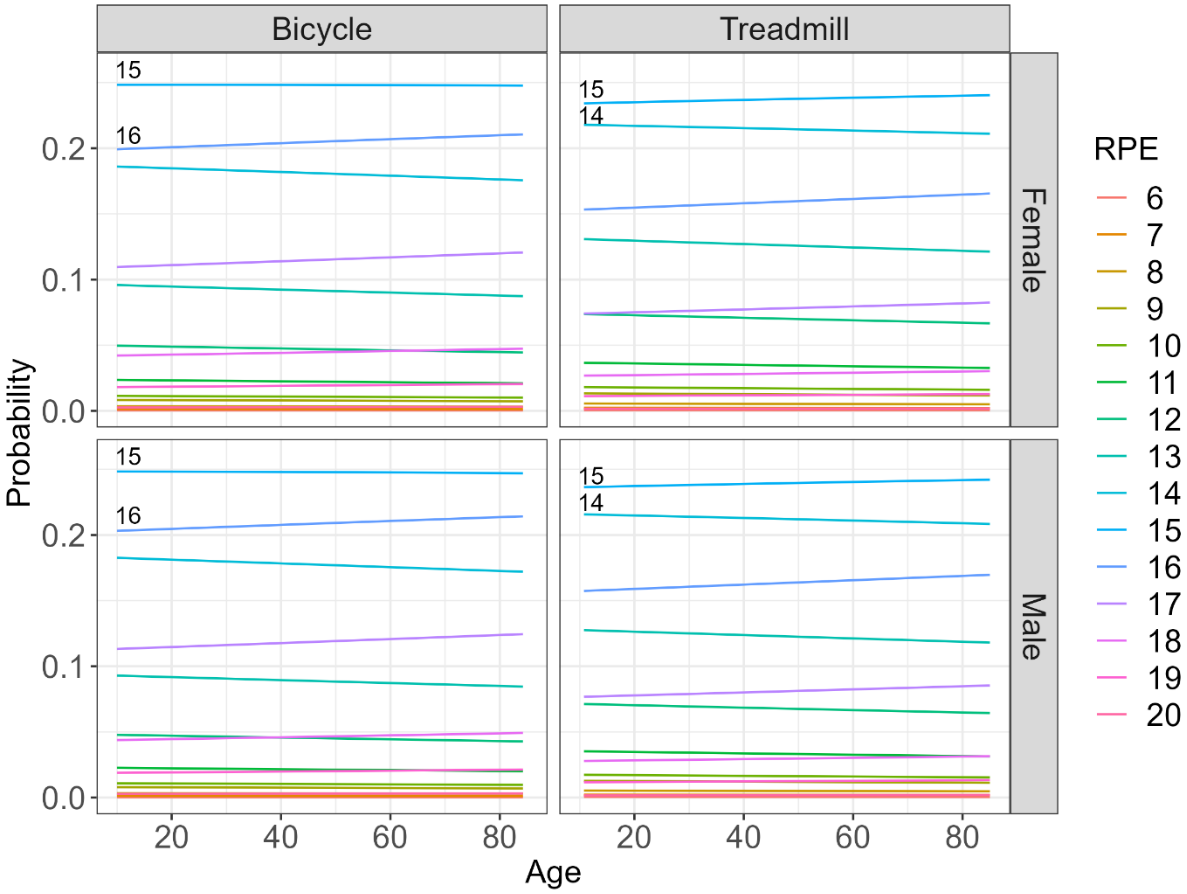

OSM 3.3 Predicted probabilities for RPE at LT2 in relation to the type of ergometry, gender and age

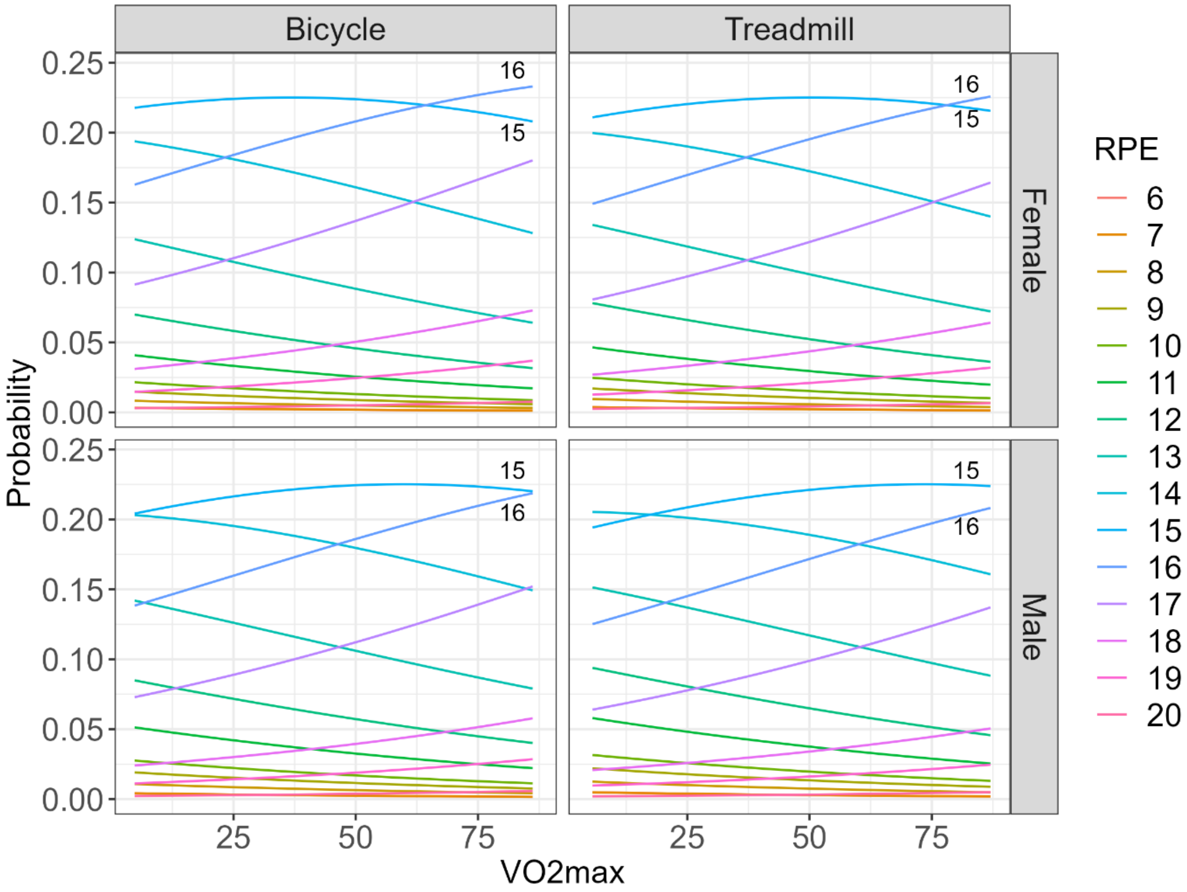

OSM 4.1 Predicted probabilities for RPE at 3 mmol/l in relation to the type of ergometry, gender and VO2max

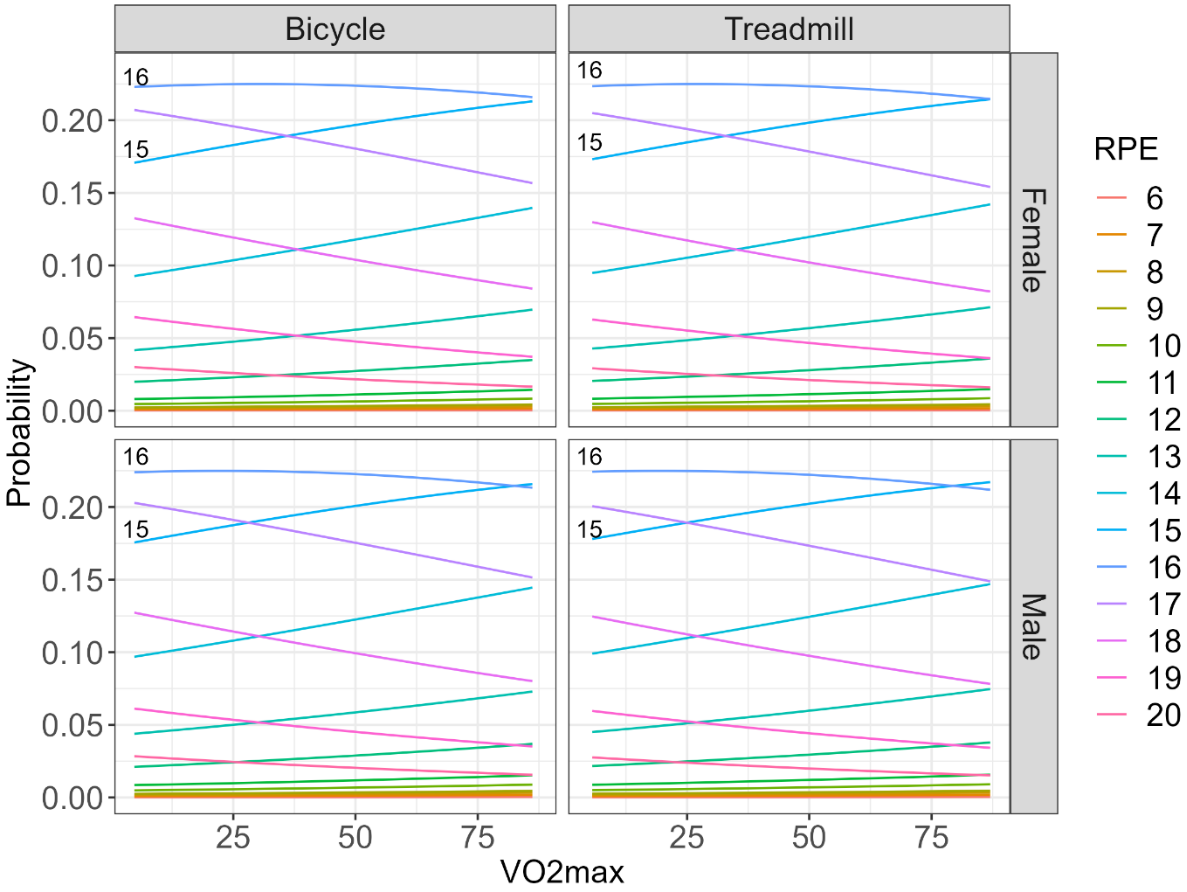

OSM 4.2 Predicted probabilities for RPE at 4 mmol/l in relation to the type of ergometry, gender and VO2max

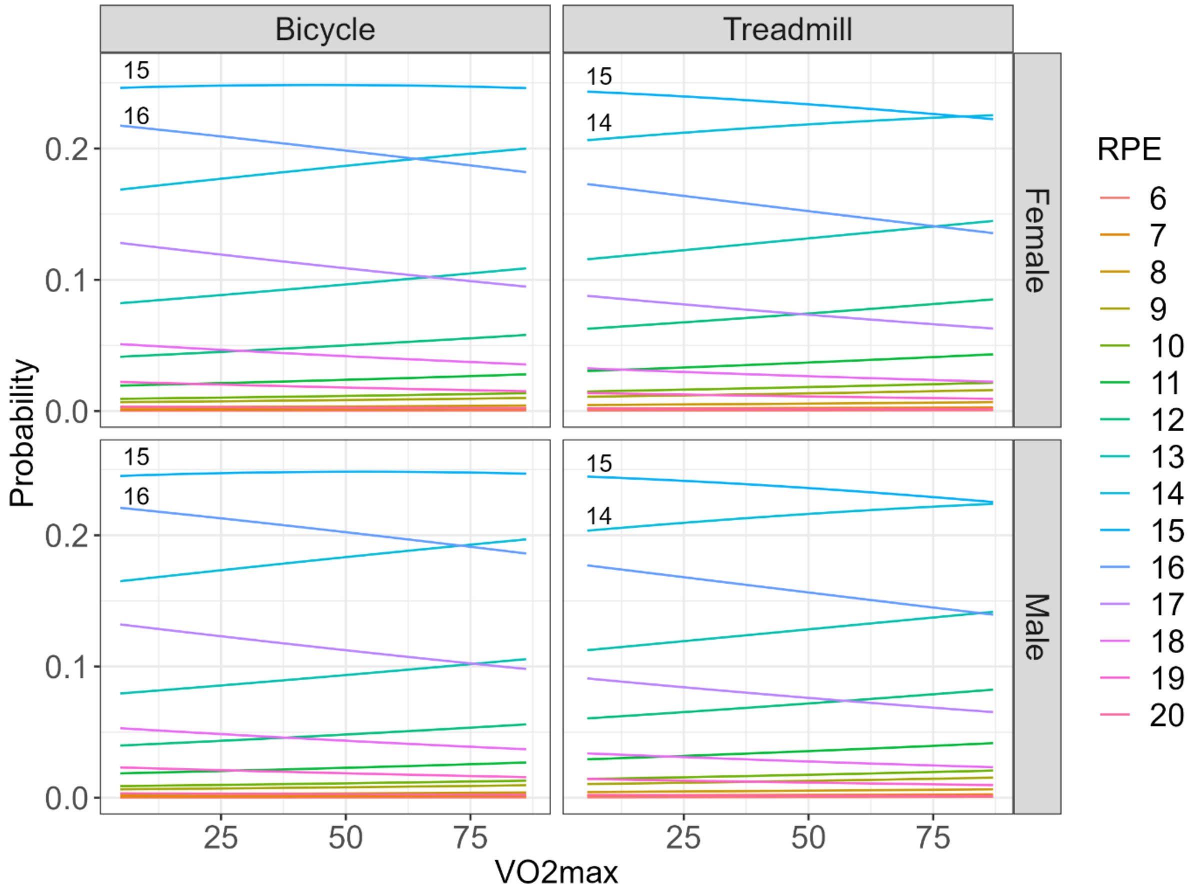

OSM 4.3 Predicted probabilities for RPE at LT2 in relation to the type of ergometry, gender and VO2max

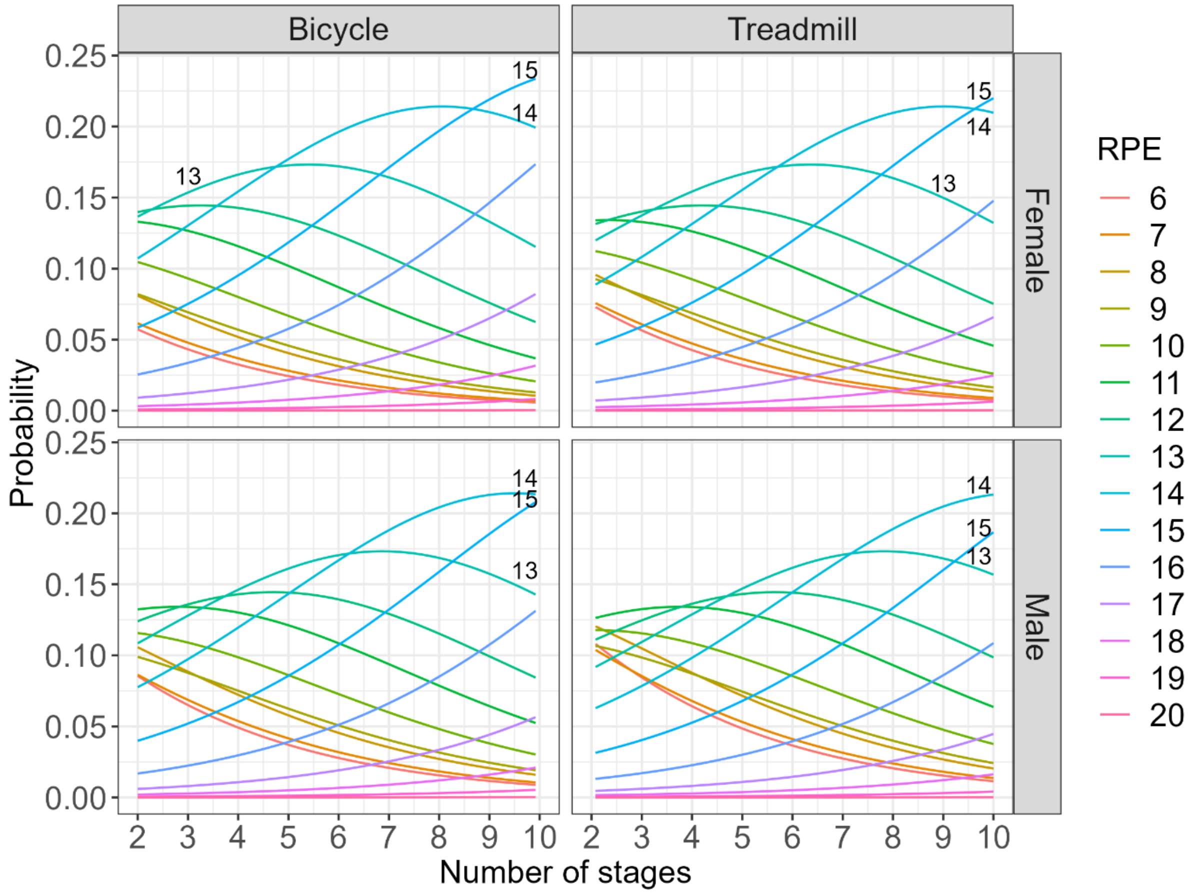

OSM 5.1 Predicted probabilities for RPE at 2 mmol/l in relation to the type of ergometry, gender and number of stages

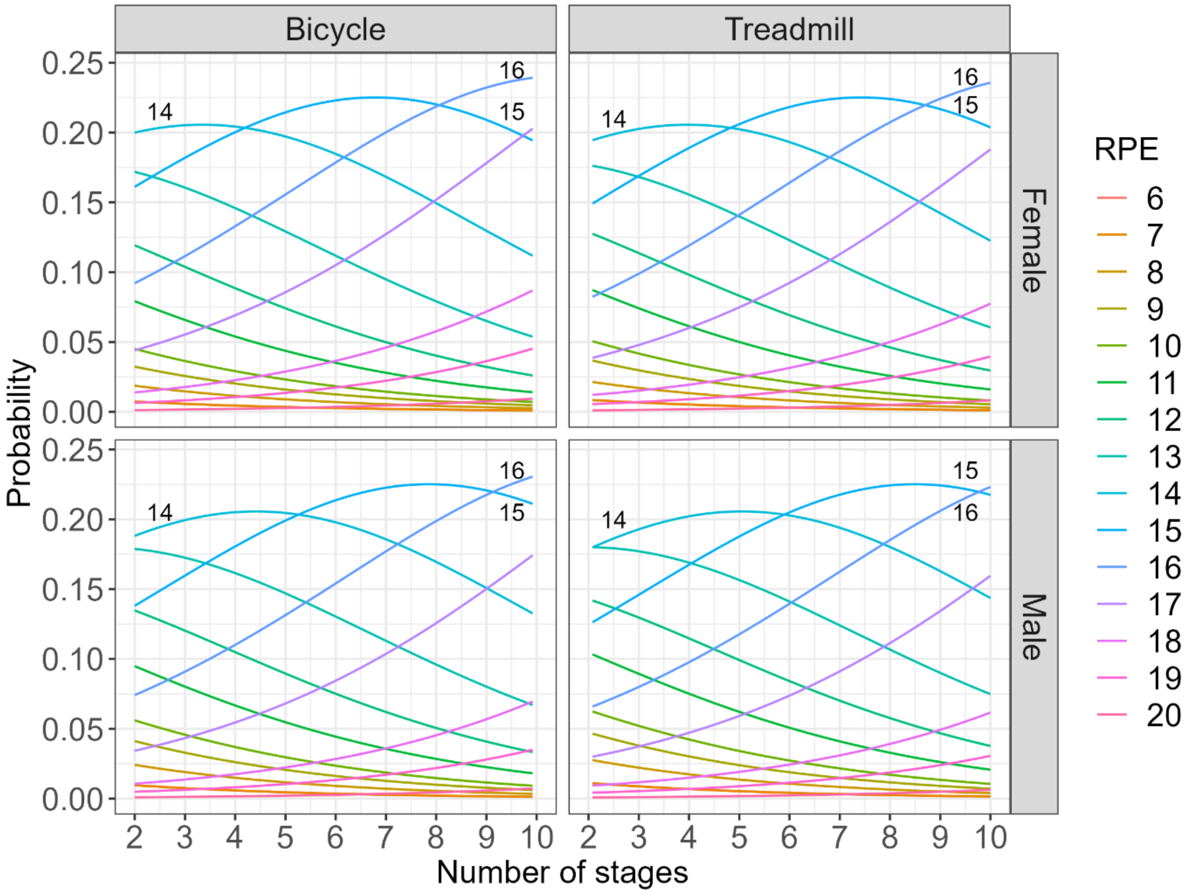

OSM 5.2 Predicted probabilities for RPE at 3 mmol/l in relation to the type of ergometry, gender and number of stages

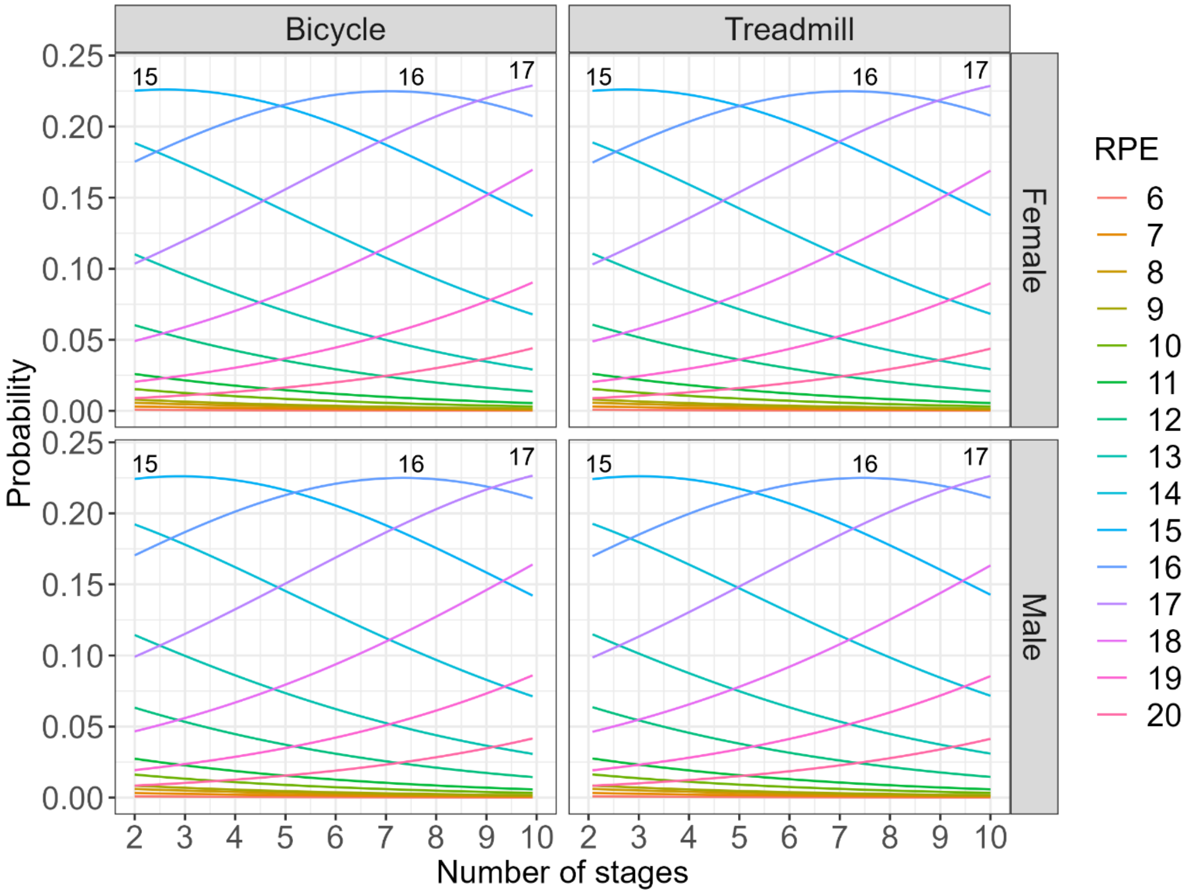

OSM 5.3 Predicted probabilities for RPE at 4 mmol/l in relation to the type of ergometry, gender and number of stages

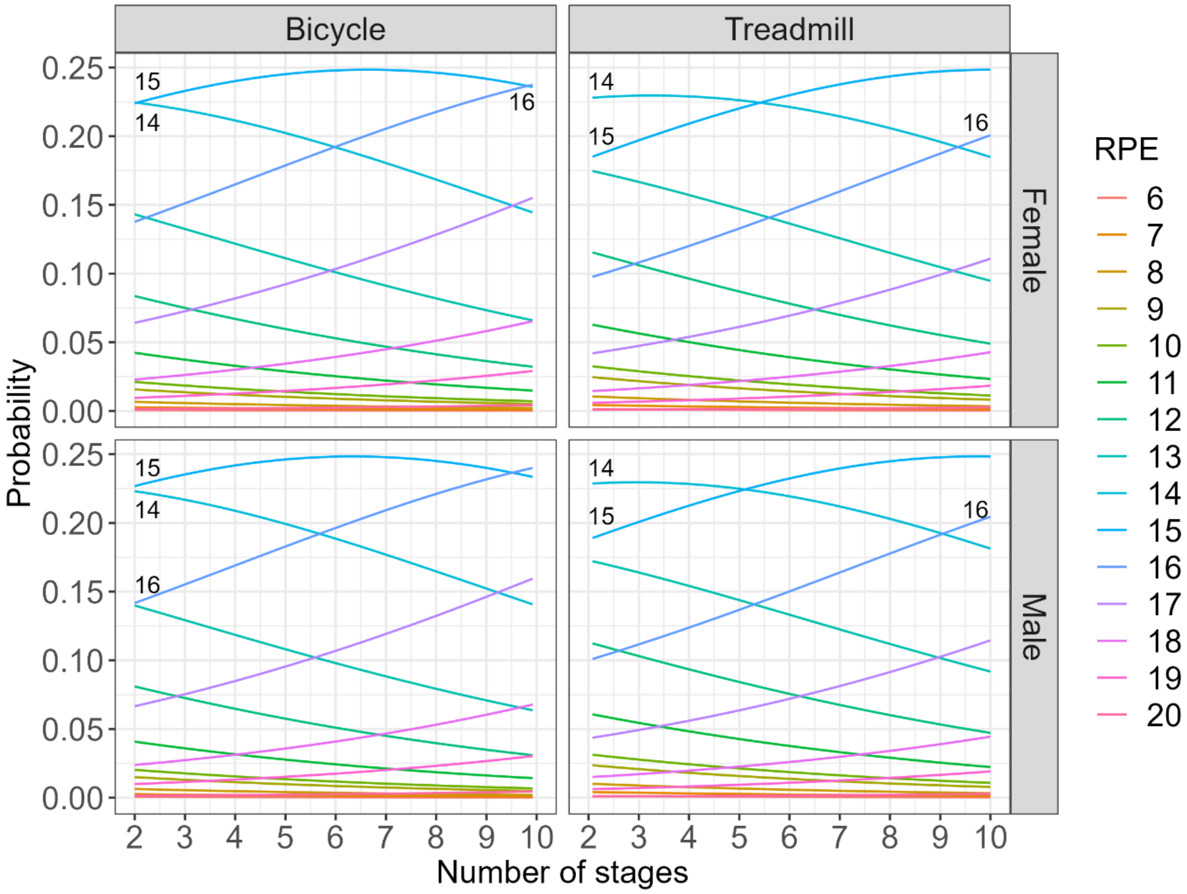

OSM 5.4 Predicted probabilities for RPE at LT2 in relation to the type of ergometry, gender and number of stages

OSM 6 R-Code

See separate R-file (R_Code_Grummt_et_al_2024.R) and corresponding R-session information (R_Session_Info_Grummt_et_al_2024.txt)
